# Repeated cobalt and chromium ion measurements in patients with bilateral large-diameter head metal-on-metal ReCap-M2A-Magnum total hip replacement

**DOI:** 10.1080/17453674.2020.1751940

**Published:** 2020-04-14

**Authors:** Sakari Pietiläinen, Heikki Mäntymäki, Tero Vahlberg, Aleksi Reito, Antti Eskelinen, Petteri Lankinen, Keijo Mäkelä

**Affiliations:** a Department of Orthopaedics and Traumatology, Turku University Hospital and University of Turku;; b Department of Orthopaedics, Tampere University Hospital and University of Turku;; c Department of Biostatistics, University of Turku;; d Coxa Hospital for Joint Replacement, Tampere, Finland

## Abstract

Background and purpose — Whole-blood (WB) chromium (Cr) and cobalt (Co) measurements are vital in the follow-up of metal-on-metal total hip replacement (MoM THR) patients. We examined whether there is a substantial change in repeated WB, Co, and Cr levels in patients with bilateral ReCap-M2A-Magnum THR. We also specified the number of patients exceeding the safe upper limit (SUL) of WB Co and Cr in the repeated measurement.

Patients and methods — We identified 141 patients with bilateral ReCap-M2A-Magnum THR operated in our institution. 61 patients had repeated WB metal ion measurements with bilateral MoM implants still in situ in the second measurement. The mean time elapsing from the first measurement (initial measurement) to the second (control measurement) was 1.9 years (SD = 0.6, range 0.2–3.5). We used earlier established SUL levels for bilateral implants by Van Der Straeten et al. (2013).

Results — The median (range) Co and Cr values decreased in the repeated measurement from 2.7 (0.6–25) to 2.1 (0.5–21) and 2.6 (0.8–14) to 2.1 (0.5–18) respectively. In 13% of the patients Co levels exceeded the SUL in the initial measurement and the proportion remained constant, at 13%, in the repeated measurement. In 5% of the patients, Cr levels were above SUL in the initial measurement and an equal 5% in the control measurement.

Interpretation — Repeated WB metal ion levels did not increase in patients with bilateral ReCap-M2A-Magnum THR with a mean 1.9-year measurement interval. Long-term development of WB metal ion levels is still unclear in these patients.

More than 20,000 metal-on-metal (MoM) hip replacements were performed in Finland during 2000–2015 (Finnish Arthroplasty Register). Currently, there are still thousands of patients with a MoM THR in situ. Whole-blood (WB) metal ion measurements are an essential part of the follow-up of MoM patients, even though they do not solely identify failing implants alone (De Smet et al. 2008, Hart et al. 2014, Reito et al. 2016).

While there is no agreed universal WB metal ion level that indicates revision surgery or predicts the outcome, different health authorities have suggested diverse follow-up protocols for the monitoring of MoM patients (Hannemann et al. 2013, MHRA 2017, US Food and Drug Administration (FDA) 2019). Furthermore, some MoM implants have better survival rates than others, which makes risk evaluation even more difficult (Matharu et al. 2016, MHRA 2017, Kasparek et al. 2018, Donahue et al. 2019).

The evaluation of patients with bilateral MoM THR is even more challenging. Patients with bilateral MoM implants often present higher levels of Co and Cr than patients with a unilateral device (Van Der Straeten et al. 2013, Reito et al. 2014, 2016). Only a few studies have assessed blood metal ion levels in patients with bilateral MoM THR. Reito et al. (2016) evaluated ion level changes in bilateral ASR THR, and ASR (DePuy, Warsaw, IN, USA) hip resurfacing arthroplasty (HRA) patients. Both WB Co and Cr were substantially higher in the ASR THR cohort in the repeated measurement (Reito et al. 2016). However, metal ion levels were not able to distinguish failing MoM components from well-functioning hips in patients with bilateral ASR THR (Reito et al. 2016, Donahue et al. 2019).

ReCap-M2A-Magnum was the most common MoM THR in Finland (Finnish Arthroplasty Register). We have previously reported that repeated metal ion measurements in unilateral ReCap-M2A-Magnum patients at a mean 2-year time interval did not show any increase (Mäntymäki et al. 2019).

We performed a retrospective comparative study to further investigate the role of repeated WB metal ion measurements in patients with bilateral M2A-ReCap-Magnum THR. Our main objectives were to investigate:

1. Is there a substantial change in the WB Co and Cr level during a follow-up period?
2. How large proportion of patients’ measurements exceed the safe upper limits (SUL) of WB Co and Cr levels in the repeated measurement (thresholds WB Co 5.0 µg/L and Cr 7.4 µg/L) (Van Der Straeten et al. 2013).

## Patients and methods

A screening program for MoM hips was launched at our institution to detect patients with adverse reactions to metal debris (ARMD). The screening was performed in consensus with the follow-up protocol recommended by the Finnish Arthroplasty Society (Finnish Arthroplasty Society 2015). The screening included anteroposterior and lateral radiographs of the hip, WB Cr and Co ion measurements, and Oxford Hip Score (OHS) questionnaire. Furthermore, if patients had poor or moderate OHS score, or elevated Cr or Co WB concentration (beyond 5 ppb), they were referred for MARS (magnetic artefact reduction sequence) MRI.

Patients with poor or moderate OHS or elevated WB ion measurements were also clinically evaluated by a senior orthopedic surgeon in an outpatient clinic. If patients had severe hip symptoms (pain, clicking, swelling) or if a pseudotumor was detected in MRI, revision surgery was considered. In addition to this, if an asymptomatic patient had WB metal ion levels above 10 ppb, revision surgery was considered to minimize the risk of Co poisoning. Patients who were not admitted for revision surgery were scheduled for annual or biannual visits in our outpatient clinic.

A ReCap-M2A-Magnum THR was used in 1,329 operations (1,188 patients) at our institution from 2005 to 2012. For this study we identified patients with bilateral ReCap-M2A-Magnum THR. Overall 141 patients (282 hips) had bilateral M2A-ReCap-Magnum THR. Of these 141 patients we identified 62 patients with at least 2 WB Co and Cr ion measurements. Of these, 3 patients had unilateral revision surgery during the follow-up period. 1 patient was revised due to aseptic loosening of the femoral component, and another for acute-onset infection. However, both patients still had both MoM bearing surfaces in situ after the revision surgery, and they remained in our study group. One patient was excluded because of unilateral revision surgery, where MoM bearing surfaces were converted to conventional ones. After this exclusion we had 61 bilateral (31 females) ReCap-M2A Magnum THR patients (122 hips) in our study group. The mean age of patients was 60 years (SD 9.7) at the time of the first hip arthroplasty. The mean femoral head size was 50 mm (SD = 3.4) and the mean acetabular inclination 44 degrees (SD = 6.3). The study period concerning primary operations was from 2005 to 2012. The follow-up data concerning ion measurements were collected from the patients until 2017.

All participating patients had their blood samples taken from the antecubital vein using a 21-gauge BD Vacutainer® Eclipse™ blood collection needle (Becton, Dickinson & Co, Franklin Lakes, NJ, USA). The first 10 mL tube of blood was used for analysis of standard laboratory tests such as C-reactive protein and erythrocyte sedimentation rate measurement. The second blood sample was taken in Vacuette® NH trace elements tube (Greiner Bio-One GmbH, Kremsmünster, Austria) containing sodium heparin. Cobalt and chromium analyses from whole blood were performed using an accredited method with inductively coupled plasma mass spectrometry (ICP-MS, VITA Laboratory, Helsinki, Finland in collaboration with Medical Laboratory of Bremen, Germany). The detection limit for Cr was 0.2 ppb and for Co 0.2 ppb. The intra-assay variation for WB Cr and Co were 2.2% and 2.7% and inter-assay variation were 6.7% and 7.9%, respectively.

### Statistics

61 patients with bilateral ReCap-M2a-Magnum THR met the criteria with at least 2 repeated metal ion measurements. The mean time elapsing from the first metal ion assessment (initial measurement) to the second (control measurement) was 1.9 years (SD 0.6, range 0.2–3.5). The time elapsing from the second hip replacement to the first (initial) metal ion measurement was considered as the follow-up time. Mean follow-up time from the second operation to the initial measurement was 4.7 years (1.9–9.0). Patients were divided into follow-up time interval groups according to the time elapsing from the second operation to the first metal ion assessment.

The individual change in 2 consecutive metal ion measurements from the same patient was modelled using a random coefficient model. Log-transformed ion values were used in conditional models due to positively skewed distribution of ion levels. Results are expressed as geometric means for better interpretability. SUL values for WB Co were 5.0 ppb and WB Cr 7.4 ppb as reported earlier (Van Der Straeten et al. 2013). P-values lower than 0.05 in a 2-tailed test were considered statistically significant.

The change over a 1.9-year measurement interval was calculated and plotted as frequency distributions for both metal ions separately.

### Ethics, funding, and potential conflicts of interest

The study was based on the national recommendation for systematic screening of MoM THR patients given by the Finnish Arthroplasty Society (2015). It was a register study, and the patients were not directly contacted. Therefore, approval by the local ethical committee was not needed. Data sharing is not possible. No benefits in any form have been received related directly or indirectly to this article. Outside this study, HM has received travel/accommodation expenses from DePuy Synthes. AE received research funding from Zimmer Biomet and DePuy Synthes and consultancy fees from Zimmer Biomet. AR reports personal fees from a paid lecture. SP, PL, KTM, and TV have nothing to disclose.

## Results (Table)

**Figure 1.**
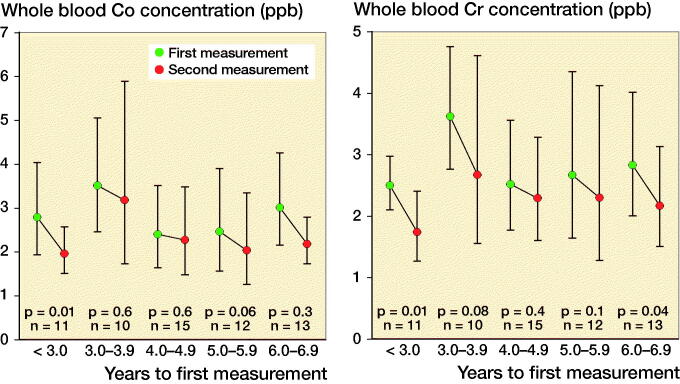
Geometric mean whole blood Co values (left) and Cr levels (right) divided across the follow-up time before initial measurement.

The geometric mean of WB Cr level decreased in the < 3-year and ≥ 6-year follow-up groups. The geometric mean of WB Co level decreased in the < 3-year group (Figure 1).

Co values were below the SUL in 49 of the 61 patients in both metal ion measurements. 4 patients (6.6%) had their Co value below the SUL in the first measurement and above the SUL in repeated measurement. Similarly, 4 patients had their Co value above the SUL in the first measurement and below the SUL in the repeated measurement. Only 4 patients had Co ion values above SUL in both measurements.

Cr values were below the SUL in 57 of the 61 patients in both metal ion measurements. Only 2 patients had their Cr value above the SUL in both measurements. 1 patient had his Cr value below the SUL in the first measurement and above the SUL in the repeated measurement. In a similar manner, 1 patient had his Cr value above the SUL in the first measurement, but below the SUL in the repeated measurement.

**Figure 2.**
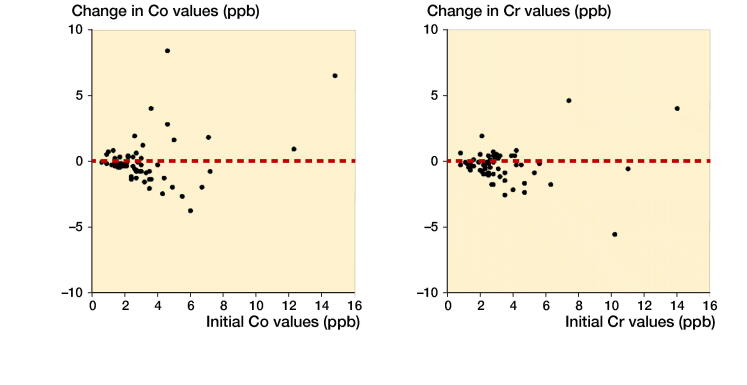
Changes in Cobalt (Co) and Chromium (Cr) ion levels compared to initial measurement

**Figure 3.**
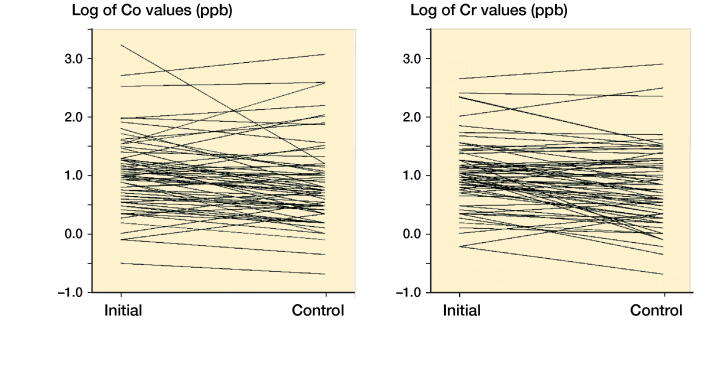
Spagetti plots for Co and Cr values at initial and control measurements. Values are naturally log-transformed.

The Co and Cr levels decreased over time and stayed mostly below the SUL if the initial value was low. The exceptions were those with high values already in the initial measurements (Figure 2). Spaghetti plots for individual Co and Cr values at initial and control measurements are presented in Figure 3. Values are naturally log-transformed.

## Discussion

The motivation for performing this study was the lack of evidence of progress of metal ion levels in bilateral ReCap Magnum THR patients.

We found that median or geometric mean WB Co and Cr levels in repeated metal ion measurements in bilateral ReCap-M2A-Magnum patients at a mean 1.9-year time interval did not show notable increase. However, our results cannot be applied to other MoM THR brands.

Data concerning ion levels of patients with a ReCap Magnum THR are scarce. A strength of our study is that we are able to present novel information, which can be used in modifying follow-up schedules worldwide. We are not aware of any other studies concerning ion levels of bilateral ReCap Magnum THRs.

A limitation of our study is that the follow-up time was short. Long-term WB ion levels in patients with bilateral ReCap-M2A-Magnum are not yet known. It is possible that a longer time range between the measurements such as 10 years might give different results. Also, the mean time interval between the WB ion measurements was only 1.9 years. Another limitation of our study is that patients with intense hip symptoms or a pseudotumor in the MRI may have been revised before any ion measurements. In addition, some of the patients who had substantially elevated WB Co or Cr levels after the initial measurement could have been admitted for revision surgery to decrease the risk of toxic effects of the metal ion levels.

The literature concerning patients with bilateral MoM hip arthroplasty is limited. Van Der Straeten et al. (2013) studied a group of 453 patients with unilateral, and 139 patients with bilateral MoM hip arthroplasty. They compared WB Co and Cr levels in patients with a well-functioning MoM hip with those who had a poorly functioning MoM hip. They suggested a SUL value of 4.6 µg/L for Cr, and 4.0 µg/L for Co in patients with unilateral MoM hip. Accordingly, they suggested SUL values of 7.4 µg/L for Cr and 5.0 µg/L for Co in bilateral patients. They stated that WB ion values above this predicted problems in metal-on-metal resurfacings. Donahue et al. (2019) proposed an even lower SUL of 4.0 µg/L for both Co and Cr for patients with bilateral ASR HRA (DePuy, Warsaw, IN, USA). The lower SUL was supposedly because ASR HRA has inferior survival to other HRA models. In their study, a SUL of 4.0 µg/L was able to successfully differentiate well-functioning implants from poorly functioning implants with a sensitivity of 42% and specificity of 90%. However, they were unable to present reliable general SUL for MoM THA due to the inadequate cohort size. In our study we used SUL values suggested by Van Der Straeten et al. (2013), because their study included also other brands in addition to ASR hip prosthesis.

The Finnish Arthroplasty Society recommends biannual metal ion measurements of MoM THA patients (Finnish Arthroplasty Society 2015). However, there are no clear guidelines on how to interpret ion concentrations and how high levels justify revision surgery. It seems that further research is needed to elucidate implant-specific WB metal ion level thresholds (Matharu et al. 2015).

Sidaginamale et al. (2013) found that metal ion concentrations are reliable indicators of abnormal wear processes in MoM implants and the Co concentration threshold of 4.5 µg/L provided good sensitivity and specificity.

Metal ion levels that should raise concern vary in different countries. In the UK, Canada and Europe values that cause alarm are between 2 ppb and 7 ppb (EFORT 2012, Health Canada 2012, MHRA 2017).

Reito et al. (2016) assessed a cohort of 76 patients with bilateral (ASR) hip resurfacings or with bilateral ASR XL THR with repeated WB ion measurements and with a median follow-up of 3.6 years. They reported no substantial difference in the HRA cohort (38 patients). However, patients with bilateral THR had a statistically significant increase in their WB Co and CR ion levels during this follow-up period (Co 8.3 µg/L vs. 12.6 µg/L, Cr 3.15 µg/L vs. 3.4 µg/L, both p < 0.001) between the 2 measurements. They therefore suggested that annual blood metal ion measurements on patients with bilateral high-risk MoM THR could be beneficial (Reito et al. 2016). We were not able to confirm this finding in patients with bilateral ReCap-M2A-Magnum THR. The poorer performance of the ASR device may explain the difference in WB ion level development compared with the ReCap Magnum THR (Seppanen et al. 2018). Matharu et al. (2017) recommended the use of different whole-blood (WB) metal ion thresholds for different implants in the follow-up MoM patients. Our current findings support this recommendation.

Mäntymäki et al. (2019) studied a group of 319 patients with unilateral ReCap-M2A-Magnum THR with repeated metal ion measurements. They had a mean follow-up time of 5.5 years (1.8–9.3) and the mean time between the measurements was 2 years. A statistically significant decrease in both Co and Cr values was detected. Both Co and Cr concentrations remained within ± 1 ppb of their initial value in the majority of patients (86% for Co, 81% for Cr). They concluded that repeated metal ion measurements may not be necessary for patients with unilateral M2A-ReCap-Magnum THR patients with WB metal ion levels below the SUL. It seems that the same may hold true even in patients with bilateral devices.

In summary, it is not necessary that patients with asymptomatic bilateral ReCap Magnum THR undergo metal ion level measurements at 2-year intervals. The optimal measurement interval is not yet known. Long-term metal ion level progression is not known either. Therefore, further research on the subject is needed.

**Table:** Differences in WB Co and Cr levels (ppb)

	Initial	Control	p-value
WB Co, n = 61			
geometric mean	2.8	2.2	< 0.007
median (range)	2.7 (0.60–25)	2.1 (0.50–21)	
WB Cr, n = 61			
geometric mean	2.8	2.3	
median (range)	2.6 (0.80–14)	2.1 (0.50–18)	< 0.001

There was a statistically significant decrease in repeated WB Co and Cr ion values.
